# Exploring Positive Survivorship Experiences of Indigenous Australian Cancer Patients

**DOI:** 10.3390/ijerph15010135

**Published:** 2018-01-15

**Authors:** Laura Tam, Gail Garvey, Judith Meiklejohn, Jennifer Martin, Jon Adams, Euan Walpole, Michael Fay, Patricia Valery

**Affiliations:** 1 School of Medicine, The University of Queensland, Herston, QLD 4006, Australia; laura.tam@uqconnect.edu.au (L.T.); jenniferh.martin@newcastle.edu.au (J.M.); 2 Menzies School of Health Research, Charles Darwin University, Darwin, NT 0811, Australia; Gail.Garvey@menzies.edu.au; 3 QIMR Berghofer Medical Research Institute, Brisbane, QLD 4006, Australia; Judith.Meiklejohn@qimrberghofer.edu.au; 4 School of Medicine and Public Health, University of Newcastle, Callaghan, NSW 2308, Australia; mikefay@me.com; 5 Faculty of Health, University of Technology Sydney, Sydney, NSW 2007, Australia; Jon.Adams@uts.edu.au; 6 Princess Alexandra Hospital, Woolloongabba, QLD 4102, Australia; Euan.Walpole@health.qld.gov.au; 7 Genesis Cancer Care, Newcastle, NSW 2290, Australia

**Keywords:** Indigenous Australians, survivorship, cancer, qualitative design

## Abstract

Amongst Indigenous Australians, “cancer” has negative connotations that detrimentally impact upon access to cancer care services. Barriers to accessing cancer services amongst Indigenous Australians are widely reported. In contrast, factors that facilitate this cohort to successfully navigate cancer care services (“enablers”) are scarcely reported in the literature. Through qualitative interviews, this article examines factors that assist Indigenous Australians to have positive cancer experiences. Semi-structured interviews were conducted with twelve adult Indigenous oncology patients recruited from a tertiary hospital in Queensland, Australia during 2012–2014. Data generated from the interviews were independently reviewed by two researchers via inductive thematic analytical processes. Discussions followed by consensus on the major categories allowed conclusions to be drawn on potential enablers. Two major categories of enablers were identified by the researchers: resilience and communication. Individual’s intrinsic strength, their coping strategies, and receipt of support improved participant’s resilience and consequently supported a positive experience. Communication methods and an effective patient-provider relationship facilitated positive experiences for participants. Despite potential barriers to access of care for Indigenous cancer patients, participants in the study demonstrated that it was still possible to focus on the positive aspects of their cancer experiences. Many participants explained how cancer changed their outlook on life, often for the better, with many feeling empowered as they progressed through their cancer diagnosis and treatment processes.

## 1. Introduction

In Australia, despite concerted efforts to “Close the Gap” [1], large socioeconomic disparities still exist between non-Indigenous and Aboriginal and Torres Strait Islander people (hereby respectfully referred to as Indigenous Australians) [2]. Health inequities are reflected in the higher morbidity and mortality rates of Indigenous Australians [3]. Unfortunately, such disparities are not isolated—with Indigenous populations across Canada, New Zealand, Japan, America, and India, all having worse outcomes than their non-Indigenous counterparts [4]. Results of targeted strategies employed internationally to minimise the health differentials have been mixed [4]. While the difference in life expectancy between Indigenous and non-Indigenous populations has reduced to 4–8 years in the USA, Canada (applicable only to the First Nation and Metis populations, excluding the Inuit population) and New Zealand [4], in Australia, the difference remains at 10.6 years, despite ongoing efforts by the federal government to address this issue [3].

Cancer is rapidly emerging as a priority health area in Australia as there is marked disparity in outcomes for Indigenous and non-Indigenous patients [5,6,7]. This may largely be attributable to receiving less treatment [8], being diagnosed later with more fatal cancers [6,7], experiencing higher rates of comorbidities [7], barriers to accessing cancer services, and the differences in which Indigenous Australians perceive the significance of their cancer diagnoses [9,10,11,12,13]. Further to this, a recent study investigating the unmet supportive care needs of Indigenous cancer patients reported at least one moderate to high unmet need at diagnosis among 54% of participants, although this reduced to one-third of patients experiencing unmet needs by six months post-diagnosis [14].

Previous research has highlighted how mistrust of the health system, stigma, fear and a lack of cultural understanding have influenced Indigenous cancer patients’ engagement in the health system [15,16]. Although we are beginning to understand the patterns of health care of Indigenous people with cancer, the survivorship narrative has fallen short. Undoubtedly, there is a role for disseminating cancer survivorship narrative in raising cancer awareness and reducing associated stigma. Positive stories about treatment and recovery also help foster support systems for patients (e.g., transport, accommodation) and highlight the importance of good communication about support services available during treatment [17]. The aim of this research was to explore the cancer care experiences of Indigenous Australians diagnosed with cancer. This paper reports on factors that assisted participants to have positive cancer experiences.

## 2. Materials and Methods 

### 2.1. Recruitment and Sampling of Patients

The recruitment of Indigenous cancer patients occurred at a large tertiary public hospital in Queensland from June 2012 to January 2014. Recruitment was conducted over a long period as the study formed a Master’s student thesis undertaken in a part-time capacity. The hospital’s Indigenous Liaison Officers provided support to the researchers in the ascertainment of participants and recruitment process through establishing rapport with the patients and extending an invitation to participate in the study. The inclusion criteria included: age >18 years, able to provide consent, have a confirmed diagnosis of any type of cancer, and either actively receiving treatment or had completed treatment for their cancer. The National Coalition for Cancer Survivorship definition of survivorship was used in this study: “An individual is considered a cancer survivor from the time of diagnosis, through the balance of his or her life” [18].

### 2.2. Interviews

Semi-structured interviews were conducted at a time and venue agreed upon between participant and researcher. Written consent was obtained from each participant prior to the interviews, with each being audio-taped for data collection. To recompense participants for their time and travel costs, a gift voucher was provided on completion of the interview. Each interview was conducted with only one interviewer and one interviewee, although for the data collection stage, there were two interviewers (Laura Tam and Judith Meiklejohn) who were unknown to the participants prior to the interview.

The interviews utilised mostly open-ended questions, thereby allowing for participants to reflect on their experiences and share their cancer experiences. Although the direction of the interviews was largely determined by the participants, topics that were covered included: supports, relationship with the health care staff, decision making, and impact of diagnosis on family and self (Table 1).

### 2.3. Data Analysis

Interviews were de-identified using a fieldwork number. Audiotapes of interviews were then transcribed verbatim. Interpretation of data was undertaken through the adoption of an inductive thematic analysis approach, whereby the qualitative data is organised inductively to explore patterns in the participants’ experiences, behaviours, and decisions [19]. Comprehensive lists of quotes relating to positive experience were grouped under categories prior to multiple face-to-face discussions between the researchers to reach consensus of the themes and categories that were identified. This process was facilitated by the graphical display of the themes and subthemes on a whiteboard, using concept maps to depict the inter-relationships between categories and to consolidate the concepts identified [20].

### 2.4. Reliability/Validity 

Reliability of the analytical process was achieved through independent analysis of the interview transcripts by two researchers (Laura Tam and Judith Meiklejohn) with subsequent ongoing discussions between members of the team (Laura Tam, Gail Garvey, Jon Adams, Jennifer Martin, and Patricia Valery) until consensus was reached on all themes. Internal validity of this study was ensured through the systematic comparison of existing data with new data to ensure that all of the themes were accounted for (Laura Tam, Gail Garvey, and Patricia Valery). The consolidated criteria for reporting qualitative research were used to guide the reporting of our findings [21]. COREQ is a 32-item checklist for explicit and comprehensive reporting of qualitative studies (Refer to Supplementary Table S1).

### 2.5. Ethics Approval

The study was approved by the Darling Downs Hospital and Health Service (HREC/12/QTDD/6); Human Research Ethics Committee of the NT Department of Health and Menzies School of Health Research (HREC-2012-1758) and Metro South Hospital and Health Service Human Research Ethics Committees (SSA/12/QPAH/334).

## 3. Results

Nineteen adults who identified as Aboriginal or Torres Strait Islander were invited to participate in the study; twelve, nine females, and three males, participated in this study. Reasons for not participating in the interview included the travel and time that was required to participate, two were critically ill, and others were unable to be contacted via the telephone numbers that they had provided.

Interview duration with twelve participants ranged from 14 min to 98 min. Most participants had a high school education level of year 10 or equivalent. Five participants lived in metropolitan areas, three lived in regional areas, and four lived in rural centres (Table 2). Interviews were conducted mostly at the hospital grounds where patients were recruited (*n* = 7); three were conducted in a public venue and two at the participant’s home. A partner or family member was present for three interviews.

Through analysis of transcripts, a number of recurring themes arose that helped facilitate participants to navigate their cancer experiences with optimism and positive outcomes. The main themes were summarised in Table 3 and were described in more detail below.

### 3.1. Resilience 

Many participants viewed cancer as a significant life challenge, but did not view the diagnosis as something insurmountable. Resilience underpinned descriptions of how many approached their cancer diagnosis and treatment. While cancer diagnosis was often referred to as a “life-changing event”, many accounts were given about how inner strength, support, and coping mechanisms facilitated acceptance and recovery.

• “I’m a fighter”

The notion of being a fighter, positive and doing all possible to survive cancer was a dominant theme: “*I’m going to fight … I want to get me head into gear, I want to start working, I want to do me own work here … I want to be myself.*” (#2514) and “*You treat the cancer, not as a disease but as another entity you’ve got to battle to prove to yourself that you deserve to live.*” (#2694). Although a diagnosis of cancer is often associated with negative connotations, many participants opted for a more pragmatic approach to their diagnosis and treatments as best depicted by one participant: “*No use sitting there and saying well ‘I’ve got cancer and I’m gonna die” … “I sort of thought well ‘I’ve got it, and I’ve got to face it and get on with it if I want to live.’ So I’m a person that if anything happens I throw it over my shoulder and say ‘let’s get on with it, let’s go.*” (#2808). For some participants, retaining a sense of independence was an important aspect of their recovery: “*I like doing things on my own. I wanted to get out of there and work. I don’t want to sit around at home, doing nothing. I want to get to work and all that.*” (#2562).

• “Support from family and friends”

The most frequently mentioned facilitating factor enabling participants to navigate through their cancer treatment with optimism was the support they received from family and friends. Many participants discussed how they drew strength and motivation from their support network and attributed this to their commitment to treatment and recovery. A female participant recounted family was her reason for living, as she described: “*If I didn’t have (son), I probably would have … chucked the towel in … there were days that you feel that … but with (son), I thought, no, I just can’t leave my boy behind. I’ve got to be around for him.*” (#2805). For other participants, the diagnosis of cancer foregrounded the depth of support available to them: “*Yeah, I’m getting support from everywhere now and … it’s absolutely lovely … It makes me feel guilty because I’ve never had so much people care and … yeah everybody’s been here.*” (#2514).

For some of the participants, their cancer diagnosis revived old friendships, whilst for others, new friendships developed: “*it brought them (friends) all closer I suppose, as I said, some friends are really good and have stuck by us.”* (#2785). New friendships provided participants with strength and a shared commonality preventing them from feeling isolated: “*when the chips are down, they’re the best relationships to build … Cos they were there with the same problems.*” (#2808). Participants recounted how both practical and emotional support from friends helped to provide a sense of calm and reason during difficult times throughout cancer treatment and recovery: *“My friends from my church also have been a real strength … by coming and helping me with housework and cooking, cleaning … They’ve been such positive ones too like telling me … it’s not the end, you’ve got to keep going.”* (#2805).

• Coping with cancer

Each participant was unique in the manner in which they coped with the cancer diagnosis and treatment. However, most participants reported a desire to not dwell on their diagnosis and to do all that is necessary to overcome their cancer. 

Some participants described how they compartmentalised their cancer diagnosis and treatment or actively addressed side effects such as loss of hair to help feel more in control and empowered in their experiences: “*I don’t want to be overloaded with things … You can only deal with so much and it’s silly for people to take on too much cos it does your head in … I was happy to just go along and sit and talk to the doctor and tell me what I needed on that day or whatever … it’s like leading up to the stem cell, you know because you have to come in for five days of chemo, and I said I’m not going to worry about any other day but that day.*” (#2692) and “*It did gradually start coming out and then I decided to take action and shave it myself … it was about taking control … and not letting things like that get to you*.” (#2562).

Several participants described how faith or physical connection to land through gardening helped them to seek peace and comfort through cancer diagnosis and treatment, as participants stated: “*Because it’s the Lord’s … the Lord gives and he takes and if it’s my turn to be then I can’t fight it and stall it*.” (#0000) and Physical connection to land through gardening was key to helping a female participant find inner peace during her treatment: “*I love my gardening … it’s the islander that comes out in me, I go out and talk to my plants, I give my bananas a kiss and hug the tress … it gives (me) that inner strength and inner peace*.” (#2805).

Some participants were able to draw on the emotional strength they had gained from other life experiences, and as a result, were able to see that their cancer diagnosis was not necessarily a death sentence. One female participant commented: “*I had cancer nine years ago and now I’m doing another treatment and you know so it was like, oh there is a light at the end of the tunnel, it’s not just a full stop and that’s it, no*.” (#2805). However, emotional resilience was not limited to past experiences with cancer, as illustrated by a female participant, who reflected upon personal tragedies to provide her with fortitude when approaching her cancer diagnosis and treatment: “*A lot of people have worse things … I’ve got really nothing to whinge about……why should I sit and dwell … feel sorry for meself …*” (#2692). Several participants had previous experiences with friends or relatives being diagnosed with cancer and these experiences had a significant role in shaping how participants cope with their own cancer diagnoses: “*I don’t know how I’d handle it but well, after seeing my mum go through all that; nothing that happens to me can compare … I knew I had cancer, I knew what I had to do to get it fixed and what else can I say or do … If I hadn’t have watched my mother die and go through all that pain then I probably would’ve been an emotional mess*.” (#2695).

As “survivors” of their own cancer experiences, many participants expressed a strong desire to be patient advocates or to help others. Much of this drive for advocacy or desire to help others largely stemmed from the uncertainty encountered by patients as they navigated through their own experiences: “*I wanted to able to be like an advocate for some other young person because I tell you what—when I went through this journey I had no idea what was in store for me*.” (#2693).

Surviving cancer also provided participants with a new purpose in life. It provided perspective and allowed participants to have the courage to pursue their own dreams and focus on the future: “*there’s been a real change because now I can look at life in a more positive angle … I always wanted to do voluntary work … I’d like to do something on the nature of helping cancer patients and so that’s my purpose of doing something like that*.” (#2805); “*But I’ve learnt one thing—if you want to do something, do it. Don’t put it off … do it because you don’t know, you could end up with a serious problem, you may not get to see it*.” (#2774); and “*It’s just a wakeup call that you know … just be thankful that you’re going to be alright and it could be a lot more worse and there’s a lot worse people out there than you, than me, you know*.” (#2693).

### 3.2. Communication and Relationships

Relationships between participants and health care professionals were noted to be highly influential in shaping the participants’ views of cancer experiences. Such relationships were not isolated to those with medical professionals but extended to relationships with the nursing staff and allied health personnel.

• “The art of communication”

A number of participants reported the importance of effective communication with health professionals to ensure that they were well informed to facilitate decision-making. Participants also described the important role effective communication plays in being aware and understanding of diagnosis and treatment, as one participant said “*The nursing staff, I can’t praise them enough. They explain everything, all the time, what’s gonna happen, what to look out for, how to look after myself if I got knocked or scratched or even like that, they went out of their way to help*.” (#2694). While effective and clear communication and feeling informed was emphasised by many some participants described relying on the health professional judgement for major decisions: “*I let the doctors make the decision to a certain degree, but I do like to know what they are doing to me. I don’t want to just be saying—you’re just a bit of meat, man-handled and to go on your merry way. I like them to tell me what they are actually doing*.” (#2774).

The importance of using clear and plain language was emphasised as a participant said: “*They made it simple and clear to me*” (#2514). While many relied on the health professional to instigate and provide appropriate information, a small number of participants described actively seeking information and how this facilitated and supported their care, as well as providing a sense of control over their treatment and recovery: “*Any questions that I had I wrote on the side of (booklet) so when I came and saw the doctor … I’d say … I didn’t understand this here … then they’d explain it to me … so I knew what I was being treated for and what the cancer was and the treatment they were going to give me was, now I could understand it*.” (#2805). 

• “Patient-provider relationships: More than just a number”

For many participants, the compassion and empathy of health professionals left long-lasting positive impressions on their cancer experiences, and thus, highlighted the importance of good relationships between patient and health professionals. Participants recounted stories of personalised care and dedication of the health staff to ensure that all individual needs and concerns were addressed. This is reflected in the following comment from a participant: “*Everybody there greeting you and asking how you were. They all know you by name and they’d ask you how your day was going and any problems you had, they had it fixed in no time.*” (#2785). Friendly inclusive care from health professionals was also described as important for providing reassurance and confidence as well as instigating conversations and support between patients as a participant said: “*the nursing staff … They treated as a person. Um, they would sit with me, for people … just talking to them you know reassuring them, that everything is fine and keeping an eye on them … And once you got the flood gates open and talking that was it, boom everybody would talk*.” (#2694). For a female participant, the compassion shown by her doctor during her moment of despair became a driving force for her to continue her battle as she recounted: “*I had tears running down my face and he just wiped them off for me and held my hand and he said ‘it’s not the end of it, this is the beginning of your new life.*” (#2805).

## 4. Discussion

The findings of this study lend considerable insights into the facilitating factors underpinning a participant’s positive cancer experiences. Support and a number of coping strategies were identified as being influential factors in building resilience. Effective communication and relationships with health professionals’ facilitated rapport and trust in health professionals and the care received. 

The diagnosis of cancer has been reported as a life-changing event and synonymous with uncertainty and devastation [23]; although, Indigenous participants in this study described how resilience helped overcome the difficulties of their diagnosis and treatment. Research about resilience from the perspective of Aboriginal people in Canada recognises the relationships between the individual, environment, and community, individual and collective actions, as well as collective historical identity, traditions, and adaptation over time [24]. Specifically, Indigenous resilience is situated as an inherent determination to overcome or prosper [25,26]. The role of social support in predicting resilience and more improved physical and psychological outcomes among people who have had a cancer diagnosis, as well as among the general population is well known [27,28,29]. This is important because cancer patients who express resilience have been reported as more likely to adhere to treatment [30] and experience less emotional distress and higher quality of life [30,31].

Similarly, participants in this study recounted how individual factors, past experiences, support, and use of a range of coping mechanisms helped them to confront and overcome a cancer diagnosis. This finding shows it is important to engage and foster a range of social supports for Indigenous people with cancer to develop resilience and improve overall outcomes.

This is the first study to the authors’ knowledge describing Australian Indigenous peoples’ perspectives of strategies employed to cope with a cancer diagnosis and recovery. Predominant strategies described by participants in this study were compartmentalising the cancer experience; actively addressing treatment and disease side effects; drawing on faith, spirituality and strength from past experiences; focusing on helping others; and, finding purpose in life and their future. Research shows a wide range of coping strategies that were employed by those diagnosed with cancer, and these vary across age [32], gender [33], and cancer type [34,35,36]. While little is known about coping among Indigenous people diagnosed with cancer, a recent systematic review of literature about coping among women from racial or ethnic minorities diagnosed with breast cancer found women from different ethnic backgrounds employed more positive coping strategies than white women [37]. The review reported that by having positive active coping strategies, such as having a fighting spirit and an established social support network, better long-term psychological adjustment can be attained. This is opposed to women who employed negative coping strategies, who, in turn, experienced greater distress and poorer survival. These findings highlight the importance for health professionals to be aware of and understand the needs of patients from different backgrounds. These findings also provide some evidence to support the need for tailored interventions to encourage positive coping strategies for people from Indigenous backgrounds. 

Barriers to communication between Indigenous people with cancer and health professionals, such as the use of medical jargon, lack of information, and racism has been reported [16,38]; Participants in this study described how clear communication with plain language facilitated their ability to make informed decisions and understand their disease and treatment. Participants also highlighted the importance of compassionate and personalised care in building trusting relationships with health professionals. Evidence suggests communication in the oncology setting should be patient centred; timely; clear; direct and respectful; and, information relayed in simple language to ensure a clear understanding [39,40]. Effective communication has been shown to reduce anxiety and improve patient satisfaction [40]. Effective information provision has been shown to improve health-related quality of life and reduce depression [41]. Further, patients who develop a strong relationship or alliance with their health professional may be more likely to adhere to treatment and experience greater psychosocial wellbeing [42,43].

As with any research, there are some strengths and limitations. A convenience sample was used for this study, and therefore, not all Indigenous cancer patients receiving cancer treatment at the hospital could be invited to participate. Patients that were not attending scheduled appointments and difficulties co-ordinating appointment and interview times with patients were some of the reasons for not approaching patients. Another limitation of this study was that data related to time since diagnosis was not consistently collected. Therefore, survivorship changes over time cannot be reported. A potential bias may have arisen, whereby the Indigenous patients who volunteered for the study were of more optimistic dispositions than those who declined participation, and thereby, they were more likely to describe positive cancer journeys. There were greater numbers of women than men that were included in the study that could be due to breast cancer being one of the more common cancers diagnosed. The younger cohort recruited in this study is consistent with a study by Banham et al. which reported that Indigenous Australians are more likely to develop cancer ten years earlier than their non-Indigenous counterparts. The findings in this study may therefore not fully represent perspectives of Indigenous men, older Indigenous people with cancer or with other cancer types not included here. Lastly, while it was clear that recurring themes could be elicited from the stories of the participants who were included in the study, thematic saturation cannot be guaranteed with the small sample size. Despite these potential biases that may impact on the generalizability of our findings, our findings provide a different perspective of Indigenous people’s experiences with cancer.

## 5. Conclusions

While a range of negative experiences are often quoted by Indigenous cancer patients, this study provides evidence that many patients have positive cancer experiences. The premise for this study was not to marginalise or reduce the significance of negative experiences, but to raise awareness of the need to re-enforce facilitating factors that may support Indigenous cancer patients to seek help early and to continue on the treatment pathway. Health professionals caring for oncology patients may apply the knowledge gained from this paper, such as: the need to re-enforce familial supports; encourage patients to embrace their spirituality and relevant cultural practices; and the need for individualized care; which will all assist patients as they navigate through their diagnosis and treatment process. Through recognizing facilitating factors, health professionals may be able to re-enforce these factors to assist patients to successfully cope and proceed through their proposed treatments. Future policies should aim to not only address barriers to access of cancer care for Indigenous patients, but also to reinforce facilitating factors that underpin survival. These policies should assist Indigenous people to have a more positive cancer experiences, and ultimately, better quality of life. 

## Figures and Tables

**Table 1:** Semi-structured interview guide to gauge patient experiences and facilitating factors for positive cancer experiences.

**Impact of Diagnosis**
What were your thoughts when you first received your diagnosis of cancer? How did you cope in those early days following the diagnosis? Do you feel like you had enough support in those early days? Often, patients report that their families have a tough time dealing with their diagnoses, how did your family cope with your diagnosis? Has anyone in the family taken time off work to help care for you? Has that caused any financial strain?
**Communication with Health Care Staff**
In terms of communicating with health professionals (such as doctors and nurses), have you had any difficulties? How would you describe your relationship with these health professionals? Did you feel you were able to approach them with any concerns you had? Often patients may be under multiple specialists and doctors, including their GP, and there is sometimes a concern amongst patients that there isn’t sufficient communication between the different treating teams. Do you have any thoughts about this or have you experienced anything similar?
**Decision-making**
In terms of treatment—were you given choices? How did you decide on what treatment to have? Some people say that they sometimes have a brief moment where they consider not going through with treatment, has that ever been the case with you? Did you feel well-supported in your decision making process? For example, did your family have any input in the process?
**Support**
Did you notice any changes in the family dynamics since you received your diagnosis or commenced treatment? Have you been able to openly discuss with family and friends about your diagnosis or your experiences with treatment? Who have you sought support from? Have you received any formal counselling or been offered it?

**Table 2:** Characteristics of study participants.

Characteristics	Number of Participants (*n* = 12)
**Gender**	
Female	9
Male	3
**Age (years)**	
20–44	4
45–64	3
65 or older	5
**Location of residence ^a^**	
Major City	8
Inner Regional	2
Outer Regional	2
**Highest education level**	
Primary school	1
Year 10 or equivalent	6
Year 12 or equivalent	1
Community college (Vocational Education) or University	4
**Employment**	
Full-time paid work	2
Part-time or casual paid work	3
Centrelink/Social security	5
Home Duties	1
Retired	1
**Type of cancer diagnosis**	
Breast cancer	5
Lymphoma	4
Other cancers (lung, thymus, head and neck)	3
**Treatment type ^b^**	
Surgery	8
Chemotherapy	10
Radiotherapy	6
Other (hormone therapy, stem cell transplant)	3
**Primary cancer vs. recurrence**	
Primary cancer	10
Recurrence or metastatic cancer	2

^a^ Remoteness of residence determined using the Australian Standard Geographical Classification Remoteness Areas, 2006 (ASGC) [22]; ^b^ Individual patients may have received a combination of treatments listed.

**Table 3:** Supporting quotes for major categories.

**Theme 1: Resilience**	
*Subthemes:*	**Quotes from Patients Demonstrating Subtheme**
1. I‘m a fighter	“*From the beginning, I saw the thing from a positive viewpoint. I didn’t think that it was going to end my life, I thought, “no, it’s not going to end my life, I’m going to look forward*” (#2805) “*If we look at cancer as a dark hole, it’s like this balloon that we tie to our hand, a black balloon, if we don’t move forward, the balloon will always be there. This dark hole will always be there. But if we’re moving forward, where will the balloon be? It’s behind you … so that’s how life should be. So that dark hole or balloon, leave it behind.*” (#2805) “*Everyone says I’m a fighter*” (#2562) “*you can’t be a weak person in society today*” (#2692) “*I’m a positive person and I always have been … as I’m not going to let anything burden me*” (#2692) “*You treat the cancer, not as a disease but as another entity you’ve got to battle to prove to yourself that you deserve to live*” (#2694)
2. Support from family and friends	“*Did I mention that my parents come to nearly all my appointments with us as it was easier for them to hear first-hand from the doctors what was happening or going to happen and for them to be able to ask questions. I found it all very hard to take all the information as it was very overwhelming. So it was great to have all the extra ears.*” (#2785) “*I have a lot of support and I’m very lucky to have that support … I think without it, even my friend, with the way, how positive she is … that I probably wouldn’t be at that stage you know*” (#2693) “*I’ve got me grandkids here and me daughter here. I’ve got everybody that’s around me, everything’s right*” (#2514) “*See I’ve got my dad’s cousins, they went through with cancer and that. They’ve been helping me out and everything*” (#2562)
3. Coping with cancer	“*I feel in life we are all dealing with a lot and if we could all just stop and realise that it isn’t just us but everybody that is dealing with many things. Then we, as a society, would be more understanding. We should take the time to listen and hear what people have to say as we can all still learn so much from life.*” (#2785) “*And then it’s just funny how things turn out you know that after all, you know you get one (breast) taken off … I’ve accepted that I’ve had a mastectomy and like I walk around home with no shirt on you know, I’ve accepted it*” (#2693) “*I think that’s (faith) another thing that has given inner strength*” (#2805) “*I think it also makes you more humane to what other people, because we all have our abilities and we all have our strengths but I think this has strengthened me and to be more to have fellow feeling*” (#2805) “*I said, ‘just take the whole thing off’, I don’t want to go fiddling and fussing, scraping and scratching so take the whole thing*” (#2805) “*It just makes you realise that you don’t really know … Don’t put it off … If you want to do anything in particular … do it because you don’t know, you could end up with a serious problem, you may not get to see it.*” (#2774)
4. Drawing strength from past experiences	“*I lost my partner 21 years ago and I’ve always been, to do things meself. If anything has to be done, it’s got to be done you know.*” (#2692) “* I don’t let anything, oh well, a lot of people have worse things and she had a terrible cancer and I thought well, I’ve got really nothing to whinge about you know, cos I was lucky cos .... (sister), she’s had a terrible husband and I’ve had a good life compared to her so I said well, why should I sit and swell, feel sorry for meself and she’s my inspiration now to keep going*” (#2692) “*after seeing my Mum go through all that nothing that happens to me can compare … I knew what I had to do to get it fixed and what else can I say or do. Just do it*” (#2695)
**Theme 2: Communication and Relationships**	
*Subthemes:*	
1. The art of communication	“*They made it simple and clear to me … Written info yeah, everything … they showed me, they showed me everything, everything that I could possibly have…very helpful*” (#2514) “*If I had any problems, the doctor would explain it to me anyway. It was very black and white.*” (#2774) “*I had radiotherapy on both of them. I didn’t have to have it on the right if I didn’t want to but I opted to because I thought, “best to have it done, rather than wait and see if it turns into cancer.*” (#2774) “*asked them all the questions and they answered it for me and that. I was fine with the doctors and the nurses*” (#2562) “*So these are the questions I had written down and they were beautifully answered for me you know? If it wasn’t the doctor, it might have been the breast care nurse or my councillor*” (#2805)
2. More than just a number	“*They treated (me) as a person ... They would sit with people for virtually that entire four hours, just talking to them you know reassuring them, that everything is fine and keeping an eye on them, you know just chatting sometimes.*” (#2694) “*And to be honest—what helped me too was seeing the nurses coming in with a smile on their faces—giving my medication or something. That helps a lot.*” (#0000) “*And the doctors here and even the breast care nurse here, they were fabulous ... And then the breast care nurses over at the (hospital), they made sure that they came up and said hello and see how the girls were going, you know the breast care nurses, they were really good, they were absolutely brilliant ... out of the blue they give you a call and say ‘how’re going?’ ‘yeah good’ ‘that’s great, it’s really good to hear that.’ They were really on the ball*” (#2805)

## Supplementary Materials

The following are available online at www.mdpi.com/1660-4601/15/1/135/s1, Table S1: Consolidated criteria for reporting qualitative studies (COREQ): 32-item checklist.

## References

[1] Gracey M. (2014). Why closing the Aboriginal health gap is so elusive. Int. Med. J..

[2] Donato R., Segal L. (2013). Does Australia have the appropriate health reform agenda to close the gap in Indigenous health?. Aust. Health Rev..

[3] Otim M.E., Kelaher M., Anderson I.P., Doran C.M. (2015). Priority setting in Indigenous health: Assessing priority setting process and criteria that should guide the health system to improve Indigenous Australian health. Int. J. Equity Health.

[4] Ring I., Brown N. (2003). The health status of indigenous peoples and others. Br. Med. J..

[5] Gibberd A., Supramaniam R., Dillon A., Armstrong B.K., O’Connell D.L. (2015). Are aboriginal people more likely to be diagnosed with more advanced cancer?. Med. J. Aust..

[6] Australian Institute of Health and Welfare (2017). Cancer in Australia.

[7] Moore S.P., Green A.C., Bray F., Garvey G., Coory M., Martin J., Valery P.C. (2014). Survival disparities in Australia: An analysis of patterns of care and comorbidities among indigenous and non-indigenous cancer patients. BMC Cancer.

[8] Valery P.C., Coory M., Stirling J., Green A.C. (2006). Cancer diagnosis, treatment, and survival in Indigenous and non-Indigenous Australians: A matched cohort study. Lancet.

[9] Shahid S., Finn L., Bessarab D., Thompson S.C. (2011). “Nowhere to room ... nobody told them”: Logistical and cultural impediments to Aboriginal peoples’ participation in cancer treatment. Aust. Health Rev..

[10] Van Schaik K.D., Thompson S.C. (2012). Indigenous beliefs about biomedical and bush medicine treatment efficacy for indigenous cancer patients: A review of the literature. Ind. Med. J..

[11] Cunningham J., Rumbold A.R., Zhang X., Condon J.R. (2008). Incidence, aetiology, and outcomes of cancer in Indigenous peoples in Australia. Lancet Oncol..

[12] Newman C., Butow P., Knight R., McMillan K., Treloar C., Kippax S., Eades S. (2008). Cancer and aboriginal people in Australia: A review of the literature. Crit. Public Health.

[13] Newman C.E., Gray R., Brener L., Jackson L.C., Johnson P., Saunders V., Harris M., Butow P., Treloar C. (2013). One size fits all? The discursive framing of cultural difference in health professional accounts of providing cancer care to Aboriginal people. Ethn. Health.

[14] Valery P.C., Bernardes C.M., Beesley V., Hawkes A.L., Baade P., Garvey G. (2017). Unmet supportive care needs of Australian Aboriginal and Torres Strait Islanders with cancer: A prospective, longitudinal study. Support. Care Cancer.

[15] McGrath P., Holewa H., Ogilvie K., Rayner R., Patton M.A. (2006). Insights on Aboriginal peoples’ views of cancer in Australia. Contemp. Nurse.

[16] Shahid S., Finn L.D., Thompson S.C. (2009). Barriers to participation of Aboriginal people in cancer care: Communication in the hospital setting. Med. J. Aust..

[17] Thompson S.C., Shahid S., Greville H.S., Bessarab D. (2011). “A Whispered Sort of Stuff” A Community Report on Research around Aboriginal People’s Beliefs about Cancer and Experiences of Cancer Care in Western Australia.

[18] Denlinger C.S., Carlson R.W., Are M., Baker K.S., Davis E., Edge S.B., Friedman D.L., Goldman M., Jones L., King A. (2014). Survivorship: Introduction and definition. Clinical practice guidelines in oncology. J. Natl. Compr. Cancer Netw..

[19] Braun V., Clarke V. (2006). Using thematic analysis in psychology. Qual. Res. Psychol..

[20] Corbin J.S., Strauss A. (2008). Basics of Qualitative Research: Techniques and Procedures for Developing Grounded Theory.

[21] Tong A., Sainsbury P., Craig J. (2007). Consolidated criteria for reporting qualitative research (COREQ): A 32-item checklist for interviews and focus groups. Int. J. Qual. Health Care.

[22] Australian Institute of Health and Welfare (AIHW) (2011). Australian Statistical Geography Standard (ASGS).

[23] Zebrack B.J. (2000). Cancer survivor identity and quality of life. Cancer Pract..

[24] Kirmayer L.J., Dandeneau S., Marshall E., Phillips M.K., Williamson K.J. (2011). Rethinking resilience from indigenous perspectives. Can. J. Psychiatry.

[25] Durie M., Te Mata o Te Tau (2007). Indigenous Resilience: From Disease and Disadvantage to the Realisation of Potential. Matariki.

[26] Merritt S. (2007). An Aboriginal perspective on resilience: Resilience needs to be defined from an Aboriginal context. Aborig. Isl. Health Work J..

[27] Wills T.A., Bantam E.O. (2012). Social support, self-regulation and resilience in two populations: General population adolescents and adult cancer survivors. J. Soc. Clin. Psychol..

[28] Stumblingbear-Riddle G., Romans J.S. (2012). Resilience among urban American Indian adolescents: Exploration into the role of culture, self-esteem, subjective well-being, and social support. Am. Indian Alsk. Nativ. Ment. Health Res..

[29] Schroevers M.J., Helgeson V.S., Sanderman R., Ranchor A.V. (2010). Type of social support matters for prediction of posttraumatic growth among cancer survivors. Psycho-Oncology.

[30] Stewart D.E., Yuen T. (2011). A systematic review of resilience in the physically ill. Psychosomatics.

[31] Min J.A., Yoon S., Lee C.U., Chae J.H., Lee C., Song K.Y., Kim T.S. (2013). Psychological resilience contributes to low emotional distress in cancer patients. Support. Care Cancer.

[32] Snöbohm C., Heiwe S. (2013). Stressors, coping and coping strategies among young adults with cancer. World. J. Psycho-Soc. Oncol..

[33] Grant M., McMullen C.K., Altschuler A., Mohler M.J., Hornbrook M.C., Herrinton L.J., Wendel C.S., Baldwin C.M., Krouse R.S. (2011). Gender differences in quality of life among long-term colorectal cancer survivors with ostomies. Oncol. Nurs. Forum.

[34] Mehrabi E., Hajian S., Simbar M., Hoshyari M., Zayeri F. (2015). Coping response following a diagnosis of breast cancer: A systematic review. Electron. Physician.

[35] Lashbrook M.P., Valery P.C., Knott V., Kirshbaum M.N., Bernardes C.M. (2017). Coping strategies used by breast, prostate, and colorectal cancer survivors: A literature review. Cancer Nurs..

[36] Kasparian N.A., McLoone J.K., Butow P.N. (2009). Psychological responses and coping strategies among patients with malignant melanoma: A systematic review of the literature. Arch. Dermatol..

[37] Yoo G.J., Levine E.G., Pasick R. (2014). Breast cancer and coping among women of color: A systematic review of the literature. Support. Care Cancer.

[38] Meiklejohn J.A., Garvey G., Bailie R., Walpole E., Adams J., Williamson D., Martin J., Bernardes C.M., Arley B., Marcusson B. (2017). Follow-up cancer care: Perspectives of Aboriginal and Torres Strait Islander cancer survivors. Support. Care Cancer.

[39] Thorne S., Hislop T.G., Kim-Sing C., Oglov V., Oliffe J.L., Stajduhar K.I. (2014). Changing communication needs and preferences across the cancer care trajectory: Insights from the patient perspective. Support. Care Cancer.

[40] Rodin G., Mackay J.A., Zimmermann C., Mayer C., Howell D., Katz M., Sussman J., Brouwers M. (2009). Clinician-patient communication: A systematic review. Support. Care Cancer.

[41] Husson O., Mols F., van de Poll-Franse L.V. (2011). The relation between information provision and health-related quality of life, anxiety and depression among cancer survivors: A systematic review. Ann. Oncol..

[42] Trevino K.M., Fasciano K., Prigerson H.G. (2013). Patient-oncologist alliance, psychosocial well-being, and treatment adherence among young adults with advanced cancer. J. Clin. Oncol..

[43] Kelley J.M., Kraft-Todd G., Schapira L., Kossowsky J., Riess H. (2014). The influence of the patient-clinician relationship on healthcare outcomes: A systematic review and meta-analysis of randomized controlled trials. PLoS ONE.

