# Supplementary material for: Exploring Positive Survivorship Experiences of Indigenous Australian Cancer Patients
Source: Int J Environ Res Public Health. 2018 Jan 15;15(1):135. doi: 10.3390/ijerph15010135 (PMC5800234; doi:10.3390/ijerph15010135)
Supplement: Supplementary file 1 [file ijerph-15-00135-s001.pdf]

**Table S1.** Consolidated criteria for reporting qualitative studies (COREQ): 32-item checklist.

| | |
|---|---|
| **Domain 1: Research team and reflexivity**<br>**Personal Characteristics** | |
| 1. Interviewer/facilitator<br>2. Credentials<br>3. Occupation<br>4. Gender<br>5. Experience and training | **Ms L Tam** was born in Hong Kong and of Asian descent but immigrated to Australia in her early childhood, completing her entire schooling in Brisbane. During the conduction of the interviews, she was in her final year of her medical degree and undertaking a MPhil.<br>**Ms J Meiklejohn**, of an Australian of Anglo-Saxon heritage, has a Bachelor of Health Science: Public Health Major, and completed a Masters of Applied Science (Research) when she undertook a qualitative research project to explore lymphoedema following cancer treatment and physical activity. |
| **Relationship with participants** | |
| 6. Relationship established | Page 2 |
| 7. Participant knowledge of the interviewer | Page 2 |
| 8. Interviewer characteristics | See above |
| **Domain 2: study design**<br>**Theoretical framework** | |
| 9. Methodological orientation and theory | Page 2 (references 14 and 15) |
| **Participant selection** | |
| 10. Sampling | Page 2 |
| 11. Method of approach | Page 2 |
| 12. Sample size | Page 4 (Results) |
| 13. Non- participation | Page 10. Non-participation discussed: not all Indigenous cancer patients receiving cancer treatment at the hospital could be invited to participate; patients not attending scheduled appointments and difficulties co-ordinating appointment and interview times with patients were some of the reasons for not approaching patients. The issue of potential bias (volunteer bias) is also discussed under study limitations. |
| **Setting** | |
| 14. Setting of data collection | Page 2 |
| 15. Presence of non-participants | Page 4 |
| 16. Description of sample | Page 4 |
| **Data collection** | |
| 17. Interview guide | Table 1 |
| 18. Repeat interviews | No |
| 19. Audio/visual recording | Interviews were audio-taped. Page 4 |
| 20. Field notes | There no field notes |
| 21. Duration | Interview duration ranged from 14 to 98 minutes. Page 4 |
| 22. Data saturation | Page 10 (Discussion) |
| 23. Transcripts returned | Interviews were conducted mostly at the hospital grounds where patients were recruited. Participants |

| | |
|---|---|
| | went home after hospital admission/clinic appointment and most lived outside Brisbane. Therefore, transcripts were not returned to participants for comments. Participants were requested to contact the research team if they had any additional questions/comments following the interview. The research team's contact details were given to participant in the study information sheet. |
| **Domain 3: analysis and findings**<br>**Data Analysis** | |
| 24. Number of data coders | Two coders (page 2) |
| 25. Description of the coding tree | Page 2. Thematic analysis of data was conducted independently by two research team members. Codes were attached to stories or quotes to indicate an action or unit of meaning. These codes were then grouped together in categories to explain initial codes. Comprehensive lists of quotes grouped under categories agreed upon by the team of researchers were compiled. Multiple discussions between the researchers were then held to reach a generalised consensus of the themes and categories identified. This process was facilitated by the use of concept maps to depict the inter-relationships between categories and to consolidate the concepts identified. |
| 26. Derivation of themes | |
| 27. Software | No specific software other than WORD was use to manage the data. |
| 28. Participant checking | Refer to item 23 above. Audiotapes of interviews were then transcribed verbatim. |
| **Reporting** | |
| 29. Quotations presented | Quotations presented throughout the Results (pages 4-7) |
| 30. Data and findings consistent | yes |
| 31. Clarity of major themes | yes |
| 32. Clarity of minor themes | yes |
